# A Qualitative Study of Japanese Medical Students’ Perspectives on Clinical Practicum during Coronavirus Disease 2019

**DOI:** 10.31662/jmaj.2025-0413

**Published:** 2025-09-26

**Authors:** Tomoko Miyoshi

**Affiliations:** 1 Center for Medical Education and Internationalization, Kyoto University, Yoshida-Konoe-cho, Sakyo-ku, Kyoto-city, Kyoto, Japan

**Keywords:** COVID-19, medical students’ perspectives, professional identity formation

The coronavirus disease 2019 (COVID-19) pandemic was a global public health crisis of unprecedented scale in recent history. During this period, health care professionals working on the front lines faced immense challenges, driven by a complex mix of fear and a strong sense of responsibility. Simultaneously, medical and other health-related students were also profoundly affected. The recent qualitative study by Suzuki et al. ^(1)^ provides a crucial analysis of this internal impact, exploring ways medical students grappled with their roles as future physicians, their desire to contribute to society, and the conflict they felt over the inability to learn as they had envisioned due to restricted clinical practicums.

The authors’ work sheds light on the attitudes of Japanese medical students toward social contribution during the pandemic. The theme of “Commitment to Supporting Patients and Healthcare Teams” stands out as a particularly original contribution. It suggests a process of professional identity formation under the pressure of a crisis, offering insights into ways students internalized their roles. This research highlights the complex emotions and struggles of medical students, who, despite a strong intrinsic motivation to contribute, were held back by systemic barriers such as legal limits of students’ role and a lack of practical training. Reports from other countries corroborate this finding, showing that medical students worldwide also indicated a desire to contribute and participated in activities such as vaccination support and public health awareness campaigns. This unique environment―where all daily information, from the status of disease in their communities to the infections of those around them, was medical in nature―likely contributed to this socialization process and their identity formation ^(2)^.

Furthermore, this study reveals students’ perceptions of the changes to their learning environment. They believed there was a significant “Decrease in Direct Clinical Learning Opportunities” and recognized both the convenience and the limitations of “Online Practicums: Balancing Benefits and Drawbacks.” Students reported a marked reduction in direct patient contact and opportunities to acquire hands-on skills. This finding aligns with trends in other countries; for instance, in the United States, “away rotations” were largely discouraged during the peak of the pandemic ^(3)^. A survey in Japanese students also showed mixed feelings about the shift in clinical practicum formats; although more than 60% of fifth- and sixth-year students were satisfied, a significant 30% were not ^(4)^. The article adeptly captures the wide range of emotions students experienced in response to these changes.

Although this research offers valuable insights, it has its limitations. As a qualitative study focused on Japan, its findings may not be universally generalizable. Moreover, it cannot compare the experiences and perceptions of students during the pandemic with those of students who completed their clinical training before or after it.

This study suggests that the professional identity formation of medical students during the pandemic may differ from that of their pre-COVID counterparts. Indeed, the pandemic has influenced students’ career paths, affecting their choices of residency training hospitals and the number of future specialists in certain fields ^(5)^. This research goes beyond simply documenting changes in student awareness of social contribution and learning environments; it provides a window into the process of identity formation itself. The reduction or absence of “embedded learning”―a core purpose of clinical training―is not merely a loss of knowledge and skills. It fundamentally affects the socialization process through which students learn from role models, gain insights from clinical experience, and develop their professional identity through constant reflection. A societal disaster such as the pandemic, which drastically altered the health care landscape, had a profound effect on this professional development. The long-term identity and career trajectories of physicians who spent their student years during this period will be a compelling topic for future study.

## Article Information

### Conflicts of Interest

None
